# All‐Trans Retinoic Acid Suppresses Hepatocellular Carcinoma Progression via the CSTB/CYTB Axis

**DOI:** 10.1111/jcmm.71012

**Published:** 2026-01-23

**Authors:** Jing Sun, Jian Zheng, Weiyi Zhu, Yang Bi, Yun He

**Affiliations:** ^1^ Department of General Surgery West China Second Hospital, Sichuan University Chengdu China; ^2^ Key Laboratory of Birth Defects and Related Diseases of Women and Children (Sichuan University), Ministry of Education Chengdu China; ^3^ Department of Pediatric Research Institute Children's Hospital of Chongqing Medical University Chongqing China

**Keywords:** anticancer, ATRA, CSTB, CYTB, hepatocellular carcinoma

## Abstract

Cystatin B (CSTB) is highly expressed in hepatocellular carcinoma (HCC) tissues and serum, indicating its potential as an early diagnostic biomarker. Given the known tumour‐suppressive effects of all‐trans retinoic acid (ATRA) in solid tumours, this study investigated whether ATRA inhibits HCC progression by modulating CSTB expression. Bioinformatics analyses of databases revealed the elevated CSTB expression in HCC, correlating with poor patient prognosis. These findings were validated in human HCC tissues and HepG2 cells. Through in vitro and in vivo functional assays, ATRA treatment was shown to significantly inhibit HCC cell proliferation, migration, and invasion, concomitantly with reduced CSTB expression. Proteomic sequencing identified cytochrome b (CYTB), a core component of mitochondrial respiratory chain complex III, as a downstream target of CSTB. Further experiments demonstrated that ATRA decreases mitochondrial membrane potential, complex III activity, and cellular ATP levels, these above effects were partially reversed upon CSTB overexpression. In vivo, ATRA administration effectively suppressed subcutaneous tumour growth. Collectively, these results indicated that ATRA exerts anti‐tumour activity in HCC by targeting the CSTB/CYTB axis, thereby impairing mitochondrial function and inhibiting tumour progression.

## 1 Introduction

Hepatocellular carcinoma (HCC) accounts for 90% of primary liver cancer cases, it is the sixth most common cancer and the third leading cause of cancer‐related deaths worldwide [1]. The 5‐year survival rate for HCC is low, at only 18% [2, 3]. The primary treatment options for early‐stage HCC are surgical resection and intrahepatic transplantation. Patients with advanced HCC are ineligible for surgical intervention, making pharmacotherapy a critical treatment option [4, 5]. However, HCC exhibits inherent resistance to chemotherapy and other systemic treatment regimens. Furthermore, the prognosis of HCC is extremely poor, largely due to the high incidence of liver metastasis [6]. Therefore, there is a pressing need to explore more effective therapeutic strategies.

Cystatin B (CSTB), a member of the cystatin superfamily, functions as an inhibitor of cysteine proteases (cathepsins). It exerts inhibitory effect by forming tight complexes, thereby limiting excessive cathepsin activity [7]. Given the pivotal role of cathepsins in lysosomal proteolysis, an imbalance between CSTB and cathepsins frequently leads to impaired autophagy and is associated with various diseases, including cancer [8]. The role of CSTB in cancer is controversial. CSTB has been shown to promote tumour growth in the PyMT mouse model of breast cancer [9]. Conversely, downregulation of CSTB in gastric cancer cells was reported to promote cell proliferation and migration [10]. Furthermore, upregulation of CSTB has also been observed in ovarian clear cell carcinoma [11]. Previous studies have demonstrated CSTB overexpression in tissues from HCC patients, along with elevated serum CSTB levels in the majority of HCC patients [12, 13]. However, the specific role and underlying mechanisms of CSTB in HCC pathogenesis remain unexplored. Elucidating the molecular characteristics of CSTB may provide a viable avenue for developing novel therapeutic interventions for hepatocellular carcinoma.

All‐trans retinoic acid (ATRA), a structurally related retinoid derived from vitamin A, serves as the active metabolite of vitamin A [14]. ATRA exerts profound effects on cellular proliferation control, differentiation induction, and apoptosis in both normal cells and various cancer cells [15]. Initially applied in acute promyelocytic leukaemia (APL), ATRA‐based therapeutic strategies induce long‐term remission in approximately 80% of APL cases [16]. Subsequently, ATRA has been investigated in a broader range of solid tumours. Recent studies have demonstrated that ATRA exerts anti‐tumour effects in breast cancer [17], gastric cancer [18], colorectal cancer [19], prostate cancer [20], hepatocellular carcinoma [14], Merkel cell carcinoma [21], and neuroblastoma [22] by acting through diverse mechanisms. However, whether ATRA can inhibit HCC progression by modulating CSTB remains unclear.

In this study, we demonstrate that CSTB promotes HCC growth and reveal that ATRA inhibits HCC progression by suppressing CSTB. Furthermore, proteomic sequencing identified CYTB as a downstream target of CSTB. We show that ATRA, via CSTB inhibition, downregulates CYTB expression, ultimately impairing mitochondrial function in HCC cells.

## 2 Materials and Methods

### 2.1 Human HCC Tissue Specimens

HCC tissues and paired adjacent non‐tumour liver tissues were obtained from Chongqing Medical University. Written informed consent was obtained from each patient prior to tissue collection. Clinical staging of cancer patients was defined by certified pathologists according to standard classification systems. All procedures involving human specimens were reviewed and approved by the Institutional Review Board of Chongqing Medical University.

### 2.2 Target Gene Expression and Survival Analysis

The University of Alabama at Birmingham CANcer data analysis Portal (UALCAN) (https://ualcan.path.uab.edu/index.html) was utilised to analyse both mRNA and protein expression levels of CSTB in HCC [23]. The Gene Expression Profiling Interactive Analysis 2 (GEPIA2) database (http://gepia2.cancer‐pku.cn/) was employed for survival analysis and to analyse gene expression data from cancerous and normal tissue samples sourced from The Cancer Genome Atlas (TCGA) and the Genotype‐Tissue Expression (GTEx) projects [24].

### 2.3 Cell Culture and Treatment

The human HCC cell line HepG2 and the human immortalised hepatocyte cell line THLE‐2 were cultured in Dulbecco's Modified Eagle Medium (DMEM) (Gibco, USA) supplemented with 10% fetal bovine serum (FBS). Cells were maintained in a humidified incubator at 37°C with 5% CO_2_. For ATRA treatment, all‐trans retinoic acid (ATRA) (Sigma, USA) was prepared by dissolving in dimethyl sulfoxide (DMSO) (Sigma, USA) to generate stock solutions of varying concentrations. In all experiments, an equal volume of DMSO served as the vehicle control.

### 2.4 Cell Transfection

The PCDH‐CSTB‐RFP and PCDH‐RFP plasmids were constructed by YouBia (Changsha, China). For cell transfection, HepG2 cells were seeded in 6‐well plates at a density of 1 × 10^6^ cells per well. Upon reaching approximately 70% confluence, cells were transfected using polyethylenimine (PEI) according to the manufacturer's instructions. Specifically, 3 μg of either the PCDH‐CSTB‐RFP or PCDH‐RFP plasmid was mixed with 8 μg of PEI and applied to the HepG2 cells. After 24 h of incubation, transfected HepG2 cells were harvested for subsequent experiments.

### 2.5 Cell Viability Assay

The HepG2 cells were seeded into 96‐well plates at a density of 5000 cells per well and cultured until adherent. Following cell attachment, the cells were treated with varying concentrations of ATRA. After treatment, the medium was aspirated and replaced with fresh medium containing 10% CCK‐8 reagent (ABclonal, China). Cells were then incubated at 37°C for 1 h. Absorbance at 450 nm was measured for each well using a microplate reader.

### 2.6 Scratch Test

Cell migratory capacity was assessed using a wound healing assay. Following trypsin digestion, cells were seeded into 6‐well plates at a density of 1 × 10^6^ cells per well. Upon reaching 90% confluence, a uniform, straight scratch wound was created in each well using a sterile 1 mL pipette tip drawn perpendicularly across the monolayer. Wound closure was monitored under a microscope, and images were captured at the indicated time points.

### 2.7 Clone Formation Assay

Cells were seeded into 6‐well plates at a density of 1000 cells per well. Following cell attachment, the cells were treated with ATRA. Treated cells were then cultured in a humidified incubator at 37°C for 16 days. At the end of the incubation period, cells were washed three times with phosphate‐buffered saline (PBS), fixed with 4% paraformaldehyde for 20 min at room temperature, and stained with 0.1% (w/v) crystal violet solution (Solarbio, Beijing, China) for 30 min. Stained colonies were visualised under an optical microscope, photographed, and counted using ImageJ software.

### 2.8 Transwell Invasion Assays

Cell invasive capacity was assessed using 24‐well Transwell chambers with polycarbonate inserts featuring 8‐μm pores (Corning, NY, USA). Each insert membrane was pre‐coated with 50 μL of Matrigel basement membrane matrix (Corning, USA) and allowed to polymerise for 24 h at 37°C. Cells (1 × 10^5^) suspended in 300 μL of serum‐free medium were seeded into the upper chamber. ATRA was added to the medium according to experimental groups. The lower chamber was filled with DMEM supplemented with 30% FBS as a chemoattractant. Following 48 h of incubation at 37°C, cells were fixed with 4% paraformaldehyde for 30 min at room temperature and stained with 0.1% crystal violet for 30 min. Invaded cells were visualised, photographed under an inverted light microscope, and quantified from five random fields per insert.

### 2.9 Detection of Apoptosis

Cell apoptosis was quantified using an Annexin V‐PE/7‐AAD Apoptosis Detection Kit (MeilunBio, China) according to the manufacturer's protocol. Cells were seeded in 6‐well plates at 4 × 10^5^ cells per well. Following experimental treatments, cells were harvested, washed twice with cold phosphate‐buffered saline (PBS), and resuspended in 1 × binding buffer at a concentration of 1 × 10^6^ cells/mL. Aliquots of 100 μL cell suspension were stained with 5 μL Annexin V‐PE and 5 μL 7‐AAD. The mixture was incubated for 15 min at room temperature in the dark. Samples were diluted with 400 μL binding buffer and analysed within 1 h using a BD FACSCanto flow cytometer. Cells exhibiting Annexin V^+^/7‐AAD^−^ staining were defined as apoptotic.

### 2.10 Quantitative Reverse‐Transcription PCR (qRT‐PCR) Analysis

Total RNA was extracted using the SteadyPure Quick RNA Extraction Kit (Accurate Biology, China) according to the manufacturer's instructions. cDNA synthesis was performed using the Evo M‐MLV Mix Kit with gDNA Clean for qPCR (Accurate Biology, China). RT‐qPCR was subsequently carried out on a Bio‐Rad CFX96 Real‐Time PCR Detection System. The reaction conditions consisted of an initial denaturation at 95°C for 3 min, followed by 40 cycles of 95°C for 15 s, 60°C for 30 s, and 72°C for 30 s. Gene expression levels were quantified using the2^−ΔΔCT^ method. The primer sequences used in this study are listed in Table 1.

**TABLE 1:** Primer sequences used in the study.

Genes	Sequences of primers (5′ → 3′)	
	Forward	Reverse
CSTB	GTGAGGTCCCAGCTTGAAGA	ACTCGCAGGTGTACGAAGTC
CYTB	ATGGTAGATGTGGCGGGTTT	TCTCCGATCCGTCCCTAACA
GAPDH	TGACTTCAACAGCGACACCCA	CACCCTGTTGCTGTAGCCAAA

### 2.11 Western Blot

Total protein was extracted using RIPA Lysis Buffer (Beyotime, China) supplemented with protease inhibitor PMSF (Beyotime, China). Proteins were separated by sodium dodecyl sulfate‐polyacrylamide gel electrophoresis (SDS‐PAGE) and transferred onto polyvinylidene fluoride (PVDF) membranes. Membranes were blocked with 5% skim milk in TBS‐T (50 mM Tris–Cl, pH 8.0, 0.05% Tween 20) for 1.5 h at room temperature, followed by overnight incubation with primary antibodies at 4°C. Secondary antibodies were incubated with PVDF membranes for 2 h at room temperature. Protein bands were detected using an Enhanced Chemiluminescence Kit (ABclonal, Cat#RM02867). Relative protein expression levels were quantified using Image Lab software by calculating the ratio of target protein to internal reference protein. Antibodies and dilution ratios used are listed in Table 2.

**TABLE 2:** Types of antibodies used in the study and dilution ratios.

Antibody	Number	Dilution	Product source
CYTB	A17966	1:1000	ABclonal (China)
CSTB	A21370	1:1000	ABclonal (China)
N‐cadherin	A3045	1:1000	ABclonal (China)
E‐cadherin	A20798	1:1000	ABclonal (China)
GAPDG	A19056	1:10000	ABclonal (China)

### 2.12 Immunohistochemistry (IHC)

Antigen retrieval was performed using heated citrate‐based antigen retrieval solution (pH 6.0) for 30 min. Endogenous peroxidase activity was blocked with 4% H_2_O_2_ for 10 min. Sections were incubated with goat serum for 30 min to block non‐specific binding, followed by overnight incubation with primary antibodies. Ki67 antibody (Abclonal, Cat#A20018, 1:200 dilution) was used to assess cell proliferation. After washing, sections were incubated with secondary antibody (Abclonal, Cat#AS014, 1:500 dilution) for 1 h at room temperature. Antibody binding was visualised using DAB, and nuclei were counterstained with haematoxylin solution. Finally, stained slides were mounted and examined under a microscope.

### 2.13 Proteomics Analysis

Protein extraction was performed as previously described. The extracted samples were sent to the Mass Spectrometry Laboratory of Chongqing Medical University for proteomic analysis. The resulting data were processed and analysed by Cosmos Wisdom Company. Data were obtained from at least three biological replicates and were presented as the mean ± standard deviation (SD). Statistical significance was determined as follows: *p*‐value < 0.05 was considered statistically significant, while n.s. denoted no significant difference.

### 2.14 Mitochondrial ROS

Cellular ROS levels were measured using a Reactive Oxygen Species Assay Kit (Beyotime, Cat#S0033S). DCFH‐DA was diluted 1:1000 in PBS to a final concentration of 10 μmol/L and added to harvested cells. Cells were incubated at 37°C for 20 min. After thorough washing to remove extracellular DCFH‐DA, resulting fluorescence was measured by flow cytometry at excitation/emission wavelengths of 485 nm/530 nm.

### 2.15 Determination of ATP Levels

ATP levels in HepG2 cells were measured using an ATP Bioluminescence Assay Kit (Beyotime, Cat#S0026). Briefly, harvested cells were lysed and centrifuged at 12,000× *g* for 5 min at 4°C. ATP levels were determined by mixing 100 μL working solution with 20 μL supernatant, where the reagent catalyzes light production from ATP and luciferin, with luminescence intensity proportional to ATP concentration. Measurements were performed using a chemiluminometer.

### 2.16 Complex III Activity

Complex III activity was measured using an assay kit (Solarbio, Cat#bc3240) according to the manufacturer's instructions. Cytosolic extracts were collected and sonicated 15 times prior to the assay. Absorbance was measured at 550 nm using a microplate reader.

### 2.17 Mitochondrial Membrane Potential

Mitochondrial membrane potential was assessed using the JC‐1 Assay Kit (Beyotime, Cat#C2006). Cells washed with PBS were incubated with JC‐1 staining working solution at 37°C for 20 min. After removing excess dye by PBS washing, samples were observed under a confocal microscope with a 20 × objective. JC‐1 monomers emit green fluorescence (excitation/emission: 485/530 nm), while aggregates emit red fluorescence (excitation/emission: 525/590 nm).

### 2.18 Animal Experiments

BALB/c nude mice (4–6 weeks old) were selected for subcutaneous xenograft modelling. A total of 5 × 10^6^ CSTB‐overexpressing HepG2 cells or control HepG2 cells were injected subcutaneously into the dorsal flanks. After 1 week, 30 μM ATRA or saline containing equivalent DMSO concentration was injected into tumour sites according to experimental groups, with injections administered every 3 days. At 5 weeks, mice were sacrificed and tumours excised for volume measurement using the formula: Volume = (width^2^ × length)/2.

### 2.19 Statistical Analysis

All data were analysed using GraphPad Prism 9 software (San Diego, CA, USA). Experimental results represent three independent replicates. Group comparisons were performed using unpaired Student's *t*‐test or one‐way ANOVA. *p* < 0.05 was considered statistically significant.

## 3 Results

### 3.1 The Expression of CSTB in HCC and Survival Analysis

**FIGURE 1:**
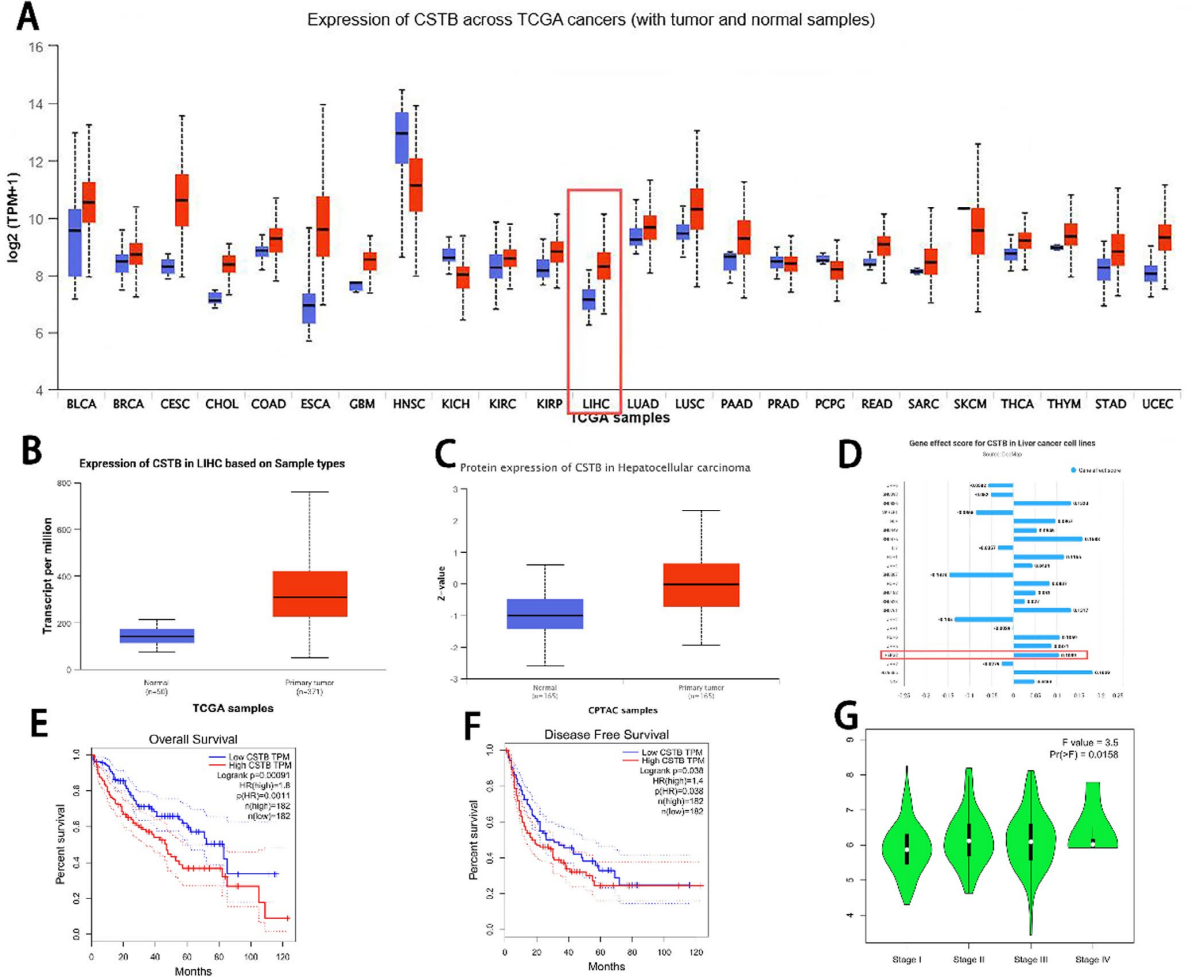
CSTB overexpression in HCC. (A) CSTB expression across cancer types analysed using UALCAN database. (B, C) CSTB mRNA (B) and protein (C) expression in normal versus HCC tissues. (D) CSTB expression profile across HCC cell lines. (E, F) Impact of CSTB expression levels on HCC patient survival analysed via GEPIA2. (G) CSTB expression stratified by HCC pathological stage.

**FIGURE 2:**
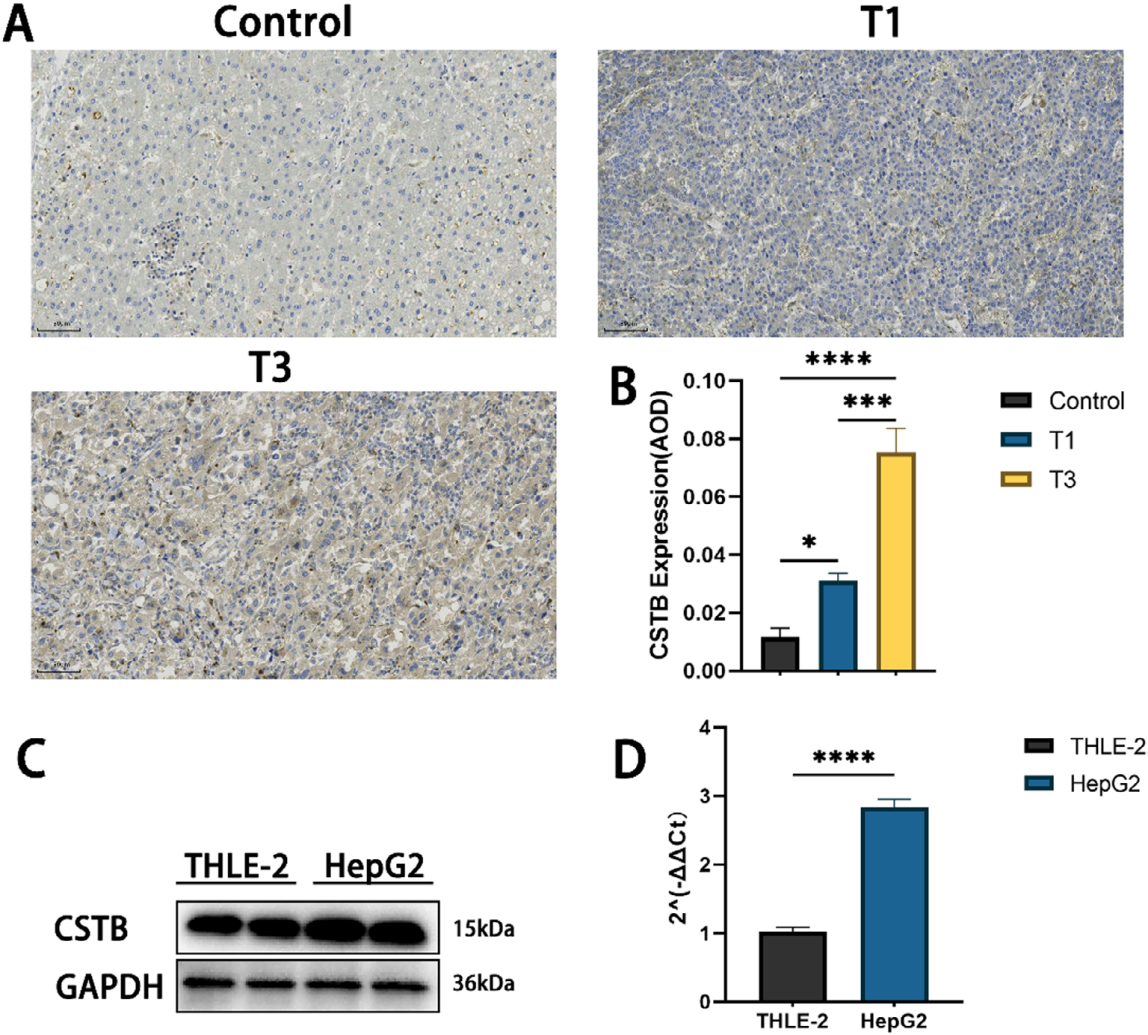
CSTB overexpression in HCC tissues and cells. (A, B) IHC staining of CSTB in normal liver tissue and HCC tissues at different pathological stages (original magnification, 20×; scale bar, 50 μm). (C, D) Protein (C) and mRNA (D) expression levels of CSTB in THLE‐2 and HepG2 cells. (*P < 0.05, ***P < 0.001, ****P < 0.0001).

To evaluate the clinical significance of CSTB, we analysed CSTB expression levels across various tumour types using the UALCAN database. Notably, CSTB mRNA and protein expression levels were significantly elevated in hepatocellular carcinoma (HCC) tissues compared to normal tissues (Figure 1A–C). Analysis of the correlation between CSTB expression and HCC pathological stage revealed a significant association between high CSTB levels and advanced cancer stage (Figure 1G). Furthermore, significant differences in Overall Survival (OS) (*p* = 0.0011) and Disease‐Free Survival (DFS) (*p* = 0.038) were observed between HCC patient groups stratified by high versus low CSTB expression (Figure 1E,F). Patients with low CSTB expression exhibited a lower mortality rate, suggesting a potential prognostic role for CSTB. Collectively, these data indicate that CSTB may play an important role in HCC progression. Subsequently, immunohistochemical (IHC) staining for CSTB was performed on pathological sections of normal liver tissue, stage T1 HCC, and stage T3 HCC, further validating these findings. CSTB protein levels were increased in both T1 and T3 stage HCC compared to normal tissue, with significantly higher levels observed in T3 stage tumours compared to T1 stage (Figure 2A,B). Additionally, analysis of the UALCAN database indicated higher CSTB expression levels in the HepG2 cell line (Figure 1D). Therefore, the human immortalised hepatocyte cell line THLE‐2 and the HCC cell line HepG2 were cultured. Western blot (WB) analysis confirmed significantly higher CSTB expression in HepG2 cells compared to THLE‐2 cells (Figure 2C,D). In summary, CSTB expression is upregulated in both HCC tissues and cell lines.

### 3.2 ATRA Downregulates CSTB Expression, Inhibiting Cell Proliferation, Invasion, and Migration

**FIGURE 3:**
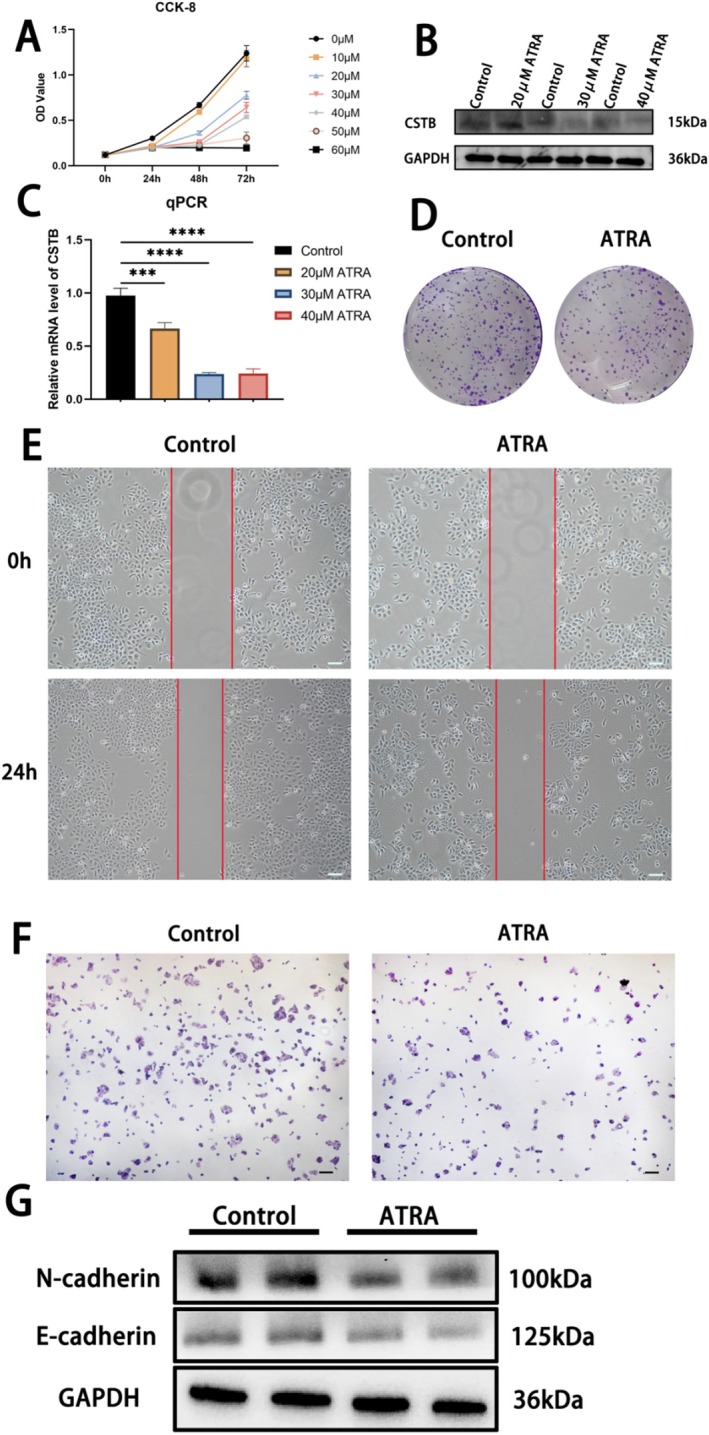
ATRA downregulates CSTB expression and inhibits HepG2 cell proliferation, invasion, and migration. (A) Cell viability of HepG2 cells treated with varying ATRA concentrations (CCK‐8 assay). (B, C) Protein (B) and mRNA (C) expression of CSTB after ATRA treatment. (D) Colony formation assay assessing ATRA's effect on proliferative capacity. (E, F) Transwell invasion assay and wound healing assay evaluating ATRA's impact on HepG2 cell metastasis in vitro (original magnification, 40×; scale bar, 100 μm). (G) Protein levels of N‐cadherin and E‐cadherin in control versus ATRA groups (GAPDH as loading control). (***P < 0.001, ****P < 0.0001).

To elucidate the impact of ATRA on HCC progression, HepG2 cells were treated with varying concentrations of ATRA (10, 20, 30, 40, 50, and 60 μM). CCK‐8 assays revealed that ATRA significantly inhibited HepG2 cell proliferation at concentrations of 20, 30, and 40 μM. However, treatment with 50 μM and 60 μM ATRA profoundly suppressed proliferation, with minimal cell growth observed (Figure 3A). WB and qPCR analyses demonstrated that CSTB expression was markedly inhibited by ATRA at concentrations of 30 and 40 μM (Figure 3B,C). Given the pronounced cytotoxic effects observed at higher ATRA concentrations, 30 μM ATRA was selected for subsequent experiments. A colony formation assay was employed to assess the effect of ATRA on long‐term proliferative capacity. ATRA treatment significantly reduced colony formation in HepG2 cells, confirming its anti‐proliferative effect (Figure 3D). The impact of ATRA on HepG2 cell migration and invasive potential was evaluated using wound healing and Transwell invasion assays, respectively. ATRA treatment resulted in a significant reduction in both migratory and invasive capabilities of HepG2 cells (Figure 3E,F). Epithelial‐mesenchymal transition (EMT) is widely recognised as a key process enabling tumour cells to acquire enhanced motility and invasiveness [25]. Consistent with the functional data, WB analysis revealed that ATRA treatment modulated EMT markers: it increased expression of the epithelial marker E‐cadherin while decreasing expression of the mesenchymal marker N‐cadherin (Figure 3G). Collectively, these results demonstrate that ATRA effectively suppresses tumour proliferation and metastatic potential in HepG2 cells.

### 3.3 CSTB Overexpression Partially Reverses the Tumour‐Suppressive Effects of ATRA

**FIGURE 4:**
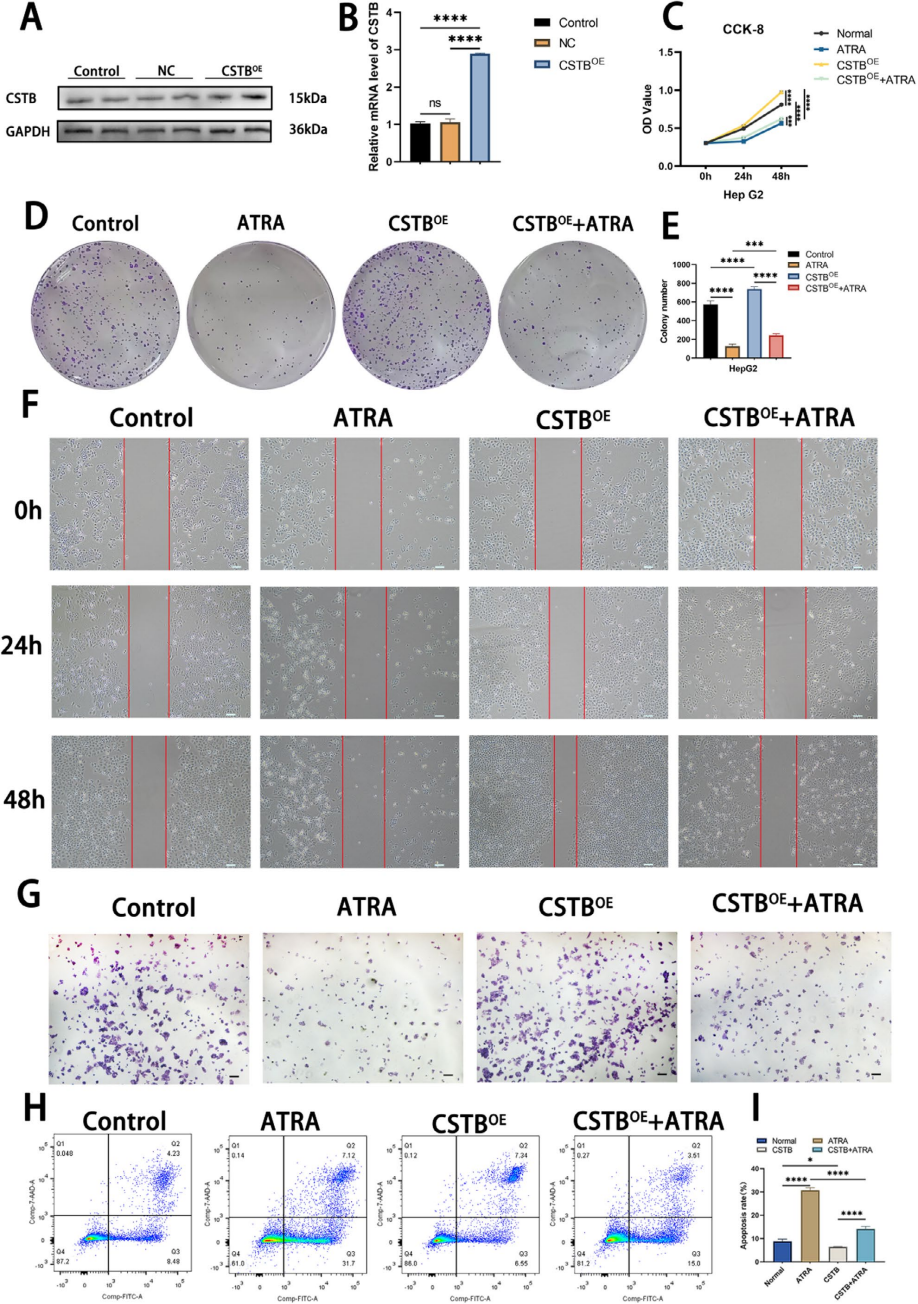
CSTB overexpression partially reverses ATRA‐mediated tumour suppression. (A) Western blot analysis of CSTB expression in control, empty vector (NC), and CSTB^OE^ groups. (B) mRNA levels of CSTB in control, empty vector (NC), and CSTB^OE^ groups. (C‐E) CCK‐8 and colony formation assays assessing reversal of ATRA‐induced proliferation suppression by CSTB overexpression. (F, G) Wound healing and Transwell invasion assays evaluating reversal of ATRA‐mediated metastasis suppression by CSTB overexpression (original magnification, 40×; scale bar, 100 μm). (H, I) Flow cytometry analysis of apoptotic reversal by CSTB overexpression following ATRA treatment. (****P < 0.0001)

To investigate whether the tumour‐suppressive effects of ATRA are mediated through CSTB downregulation, we generated CSTB‐overexpressing (CSTB^OE^) HepG2 cells. Transfection efficiency was confirmed by WB and qPCR, revealing significantly elevated CSTB expression levels in the CSTB^OE^ group compared to controls (Figure 4A,B). CSTB^OE^ HepG2 cells were then treated with 30 μM ATRA to assess whether CSTB overexpression could counteract ATRA's effects. CCK‐8 and colony formation assays demonstrated that CSTB‐OE partially restored the proliferative capacity impaired by ATRA treatment (CSTB^OE^ + ATRA group vs. ATRA group) (Figure 4C–E). Similarly, Transwell invasion and wound healing assays indicated a partial recovery of migratory and invasive capabilities in the CSTB^OE^ + ATRA group relative to the ATRA group alone (Figure 4F,G). Collectively, these findings demonstrate that CSTB overexpression partially rescues ATRA‐induced suppression of HepG2 cell proliferation, migration, and invasion in vitro. Subsequent flow cytometric analysis of apoptosis revealed that CSTB overexpression significantly reduced basal apoptosis in HepG2 cells compared to the control group. Furthermore, the apoptotic rate in the CSTB^OE^ + ATRA group was significantly lower than that in the ATRA group (Figure 4H,I). In conclusion, these results demonstrate that ATRA exerts its tumour‐suppressive effects, at least in part, through downregulation of CSTB.

### 3.4 ATRA Impairs Mitochondrial Function in HepG2 Cells via the CSTB/CYTB Axis

**FIGURE 5:**
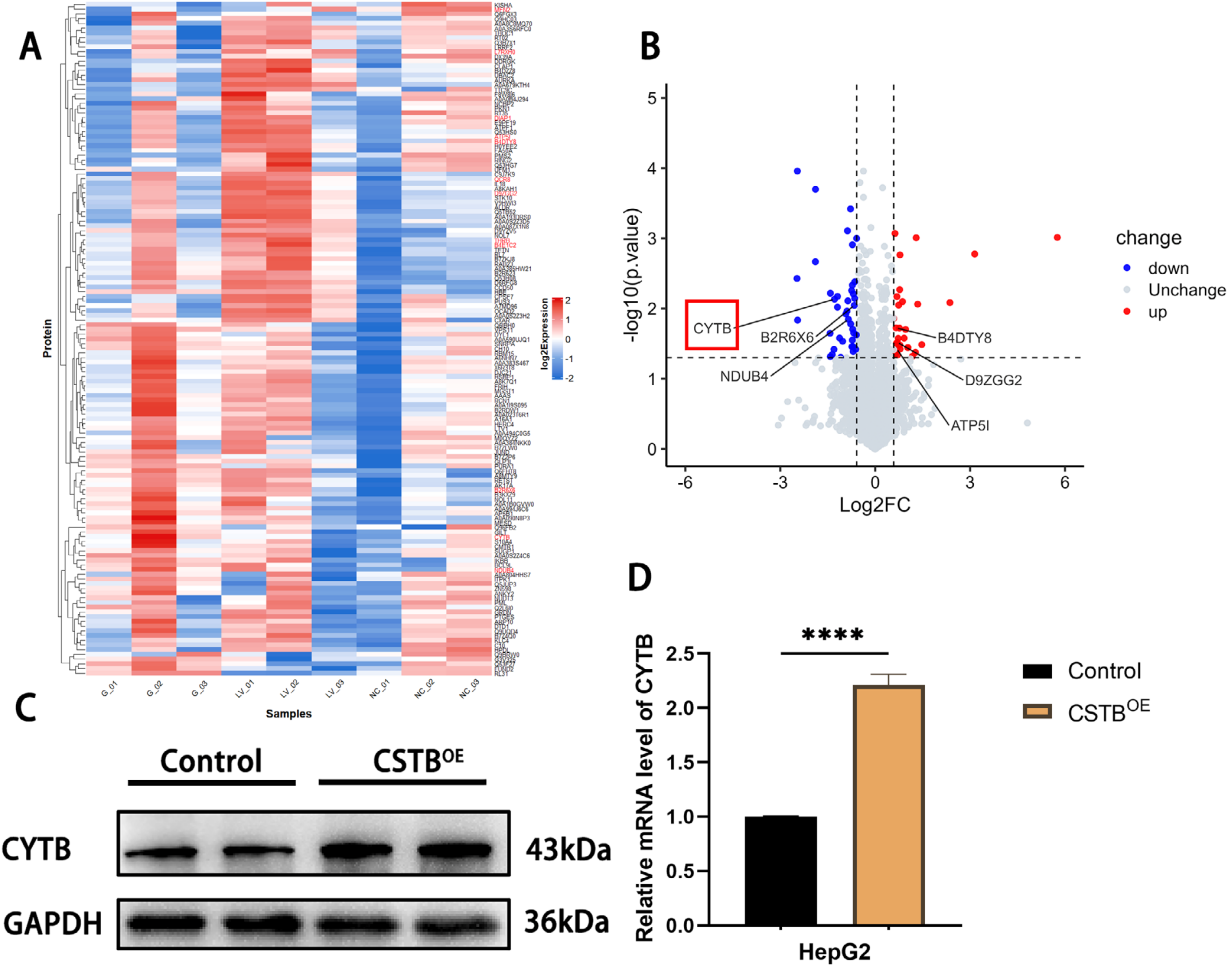
Downstream gene analysis by proteomics. (A) Heatmap of differentially expressed proteins in CSTB‐silenced HepG2 cells (LV group), negative control (NC group), and normal HepG2 cells (G group). (B) Volcano plot of differentially expressed proteins in LV vs. G groups. Red and blue dots represent proteins with increased or decreased expression, respectively, in the LV group compared to the G group. (C, D) Western blot (C) and qPCR (D) analysis of CYTB protein and mRNA expression in HepG2 cells after CSTB overexpression. (****P < 0.0001)

**FIGURE 6:**
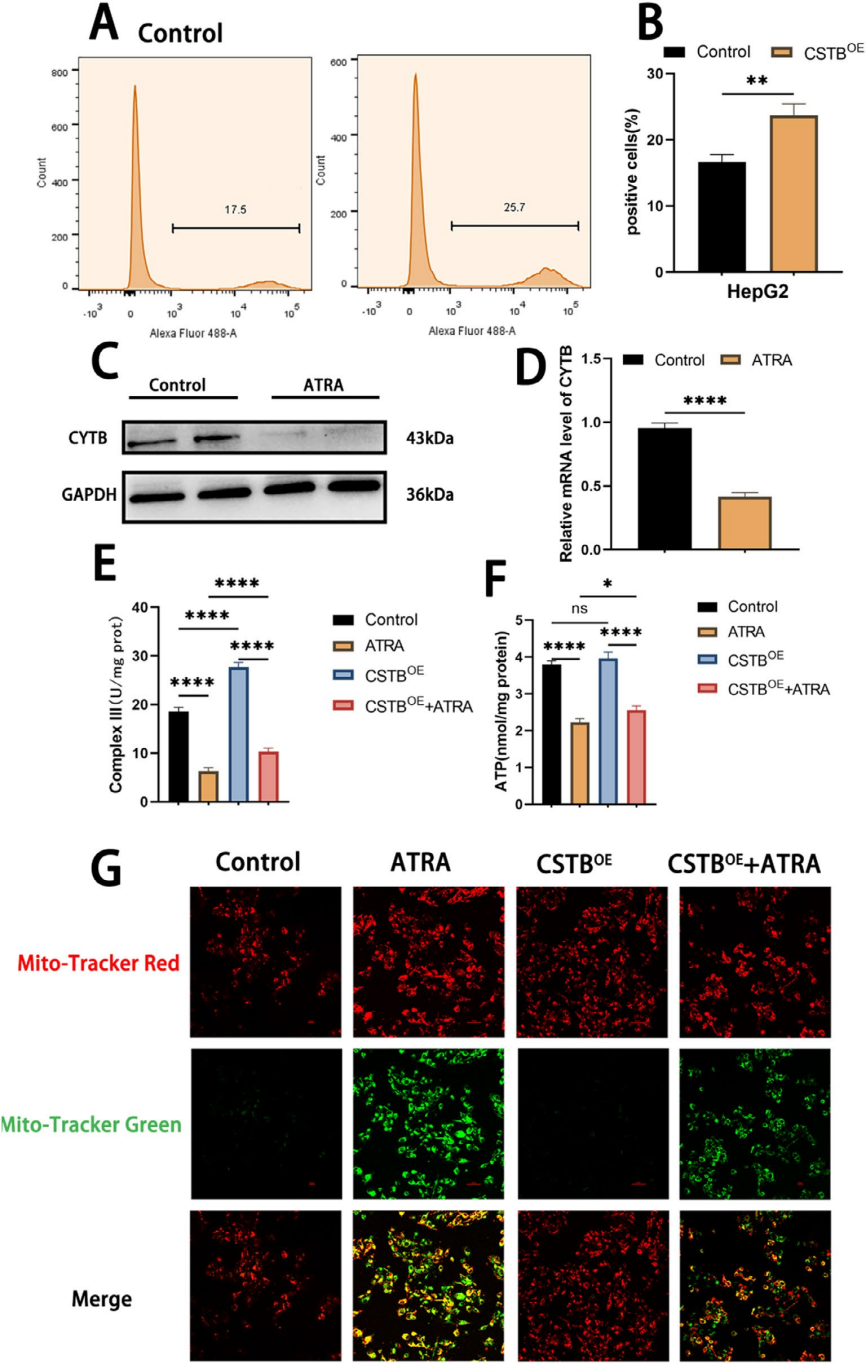
ATRA suppresses mitochondrial function via CSTB/CYTB. (A, B) Cellular ROS levels detected by flow cytometry using DCFH‐DA probe; (B) quantitative data. (C, D) Western blot (C) and qPCR (D) showing ATRA‐induced suppression of CYTB protein and mRNA expression in HepG2 cells. (E) ATP activity after ATRA treatment. (F) Mitochondrial respiratory chain complex III activity post‐ATRA treatment. (G) JC‐1 staining measuring mitochondrial membrane potential (confocal microscopy; original magnification 200×; scale bar 50 μm). Red fluorescence: intact membrane potential; green fluorescence: depolarized membrane potential (original magnification, 200×; scale bar, 50 μm). (*P < 0.05, **P< 0.01, ****P < 0.0001)

To identify downstream targets of CSTB in HepG2 cells, global proteomic sequencing was performed on CSTB‐silenced HepG2 cells (LV group), negative control cells (NC group), and normal HepG2 cells (G group). Methods for cell preparation and validation of knockdown efficiency were described previously [26]. Comparative proteomic analysis revealed 68 significantly dysregulated proteins in the LV group versus the G group. Among these, 29 proteins were upregulated and 39 were downregulated (Figure 5A). Notably, cytochrome b (CYTB) was one of the most significantly downregulated proteins in the LV group (log2(FC) = −0.387, *p*‐value = 0.00735) (Figure 5B). To further validate CYTB as a downstream effector of CSTB, WB and qPCR analyses were conducted on CSTB^OE^ HepG2 cells. Both CYTB protein and mRNA expression levels were significantly elevated in the CSTB^OE^ group compared to the control group (Figure 5C,D), confirming CYTB as a CSTB downstream target. Given that CYTB, encoded by the mitochondrial gene MT‐CYB, is an essential core subunit of mitochondrial respiratory chain complex III, we hypothesised that ATRA might impact mitochondrial function. First, intracellular reactive oxygen species (ROS) accumulation was measured using the ROS‐sensitive fluorescent probe DCFH‐DA coupled with flow cytometry. CSTB overexpression significantly increased cellular ROS levels (Figure 6A,B). This finding suggests that CSTB‐driven CYTB overexpression may promote electron leakage within the mitochondrial respiratory chain, potentially due to CYTB's role as an electron carrier in complex III, consequently leading to elevated ROS production. Subsequently, WB analysis confirmed that ATRA treatment downregulated CYTB protein expression (Figure 6C). Assessment of respiratory chain complex III activity across treatment groups demonstrated that ATRA significantly inhibited complex III activity. This inhibition was partially rescued by CSTB overexpression. A similar pattern was observed for cellular ATP levels, which were significantly reduced by ATRA and partially restored by CSTB‐OE (Figure 6E,F). Finally, JC‐1 fluorescent probes were used to further characterise the effect of ATRA on mitochondrial membrane potential (MMP). Immunofluorescence analysis revealed a significant increase in green fluorescence intensity in the ATRA group. This shift indicates dissipation of MMP in HepG2 cells following ATRA treatment. Notably, CSTB overexpression ameliorated this ATRA‐induced loss of MMP (Figure 6G). Collectively, these results demonstrate that ATRA suppresses respiratory chain complex III activity via the CSTB/CYTB axis, leading to reduced ATP production and mitochondrial dysfunction.

### 3.5 ATRA Suppresses HCC Tumour Growth In Vivo

**FIGURE 7:**
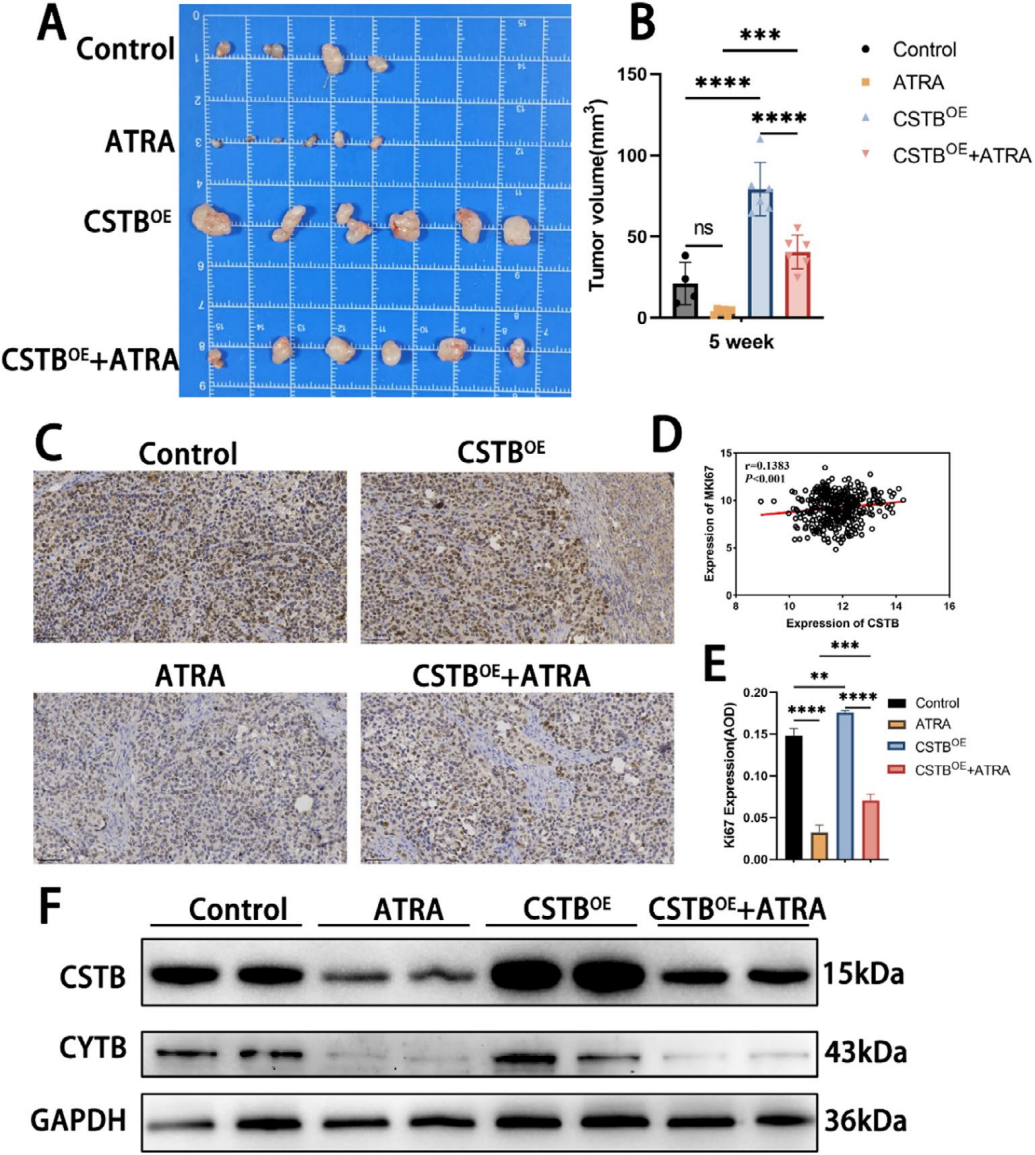
ATRA suppresses tumour growth in vivo. (A) Tumour tissues obtained from experimental groups. (B) Calculated tumour volumes. (C–E) Correlation between CSTB and Ki67 via GEPIA2 (C), Ki67 IHC staining in tumour tissues (D; original magnification 20×; scale bar 50 μm), and quantitative analysis (E). (F) Protein expression of CSTB and CYTB in tumour tissues (GAPDH loading control). (**P < 0.01, ***P < 0.001, ****P < 0.0001)

To evaluate the effect of ATRA on in vivo tumour growth, a subcutaneous xenograft tumour model was established in nude mice using HepG2 cells. Notably, tumours overexpressing CSTBOE group exhibited significantly increased volume compared to control tumours, underscoring the critical pro‐tumorigenic role of CSTB (Figure 7A,B). ATRA treatment significantly reduced xenograft tumour volume relative to the control group. However, tumour volume in the CSTBOE + ATRA group was significantly larger than in the ATRA group (Figure 7A,B). This finding indicates that ATRA also suppresses tumour growth in vivo, at least partially, through downregulation of CSTB. Analysis of the UALCAN database revealed a significant positive correlation between CSTB expression and the proliferation marker Ki67 (MKi67 in humans) in HCC samples (*r* = 0.1383, *p* < 0.001) (Figure 7D). Consistent with this, IHC staining for Ki67 in xenograft tumour sections demonstrated that ATRA significantly inhibited the proliferative activity of HepG2 cells in vivo. This anti‐proliferative effect of ATRA was partially reversed by CSTB overexpression (Figure 7C,E). Finally, WB analysis of tumour lysates confirmed decreased expression of both CSTB and CYTB in the ATRA‐treated group, while CSTB overexpression attenuated this decrease. These protein level changes were consistent with the functional and histological findings (Figure 7F). Overall, these in vivo results demonstrate that ATRA inhibits HCC progression via the CSTB/CYTB axis.

## 4 Discussion

While early diagnosis and multidisciplinary management of HCC offer significant therapeutic benefits, long‐term prognosis remains poor. Advances in gene sequencing technologies have facilitated numerous studies elucidating the roles of various genes in HCC pathogenesis and progression [13, 27, 28]. It has been recently reported the role of novel genes in driving hepatocellular carcinoma progression, such as Floittillin‐1 regulated Golgi homeostasis, NSUN2‐mediated immune evasion [29, 30, 31]. Notably, CSTB is overexpressed in the majority of HCC tissues and exhibits elevated serum levels in most HCC patients. Previous experimental evidence has consistently demonstrated dysregulated CSTB expression in association with multiple cancer types, suggesting its potential as an early diagnostic biomarker for HCC and ovarian epithelial tumours [12, 32]. Consequently, the biological function and underlying mechanisms of CSTB in HCC warrant further investigation. In the present study, we initially analysed CSTB expression in HCC using the UALCAN database. Subsequent experimental validation in HepG2 and THLE‐2 cell lines, alongside HCC patient tissues, confirmed significant CSTB upregulation in HCC tissues and cell lines. Furthermore, high CSTB levels were correlated with advanced HCC stage. Prognostic analysis via the GEPIA2 database revealed a significant association between elevated CSTB expression and poor patient survival in HCC. Gain of functional assays demonstrated that CSTB promotes HepG2 cell proliferation both in vitro and in vivo. These findings underscore the oncogenic role of CSTB in HCC and highlight its potential as a predictive biomarker for HCC patients.

ATRA, established as a first‐line therapy for APL, has also been investigated in various solid tumours [33, 34]. Prior studies demonstrated that ATRA can differentiate highly infiltrated myeloid‐derived suppressor cells (MDSCs) in HCC into mature myeloid cells, enhance anti‐tumour T‐cell responses, and significantly inhibit tumour angiogenesis and fibrosis in Hepa1‐6 cells, suggesting its potential as a therapeutic agent for hepatocellular carcinoma [35]. In the present study, guided by UALCAN database analysis, we selected HepG2 cells for investigation. ATRA significantly suppressed HepG2 cell proliferation, migration, and invasion, while concurrently promoting apoptosis. Epithelial‐mesenchymal transition (EMT) is a critical driver of cancer progression, conferring a more metastatic and invasive phenotype on tumours [36], and is closely linked to tumour recurrence [37]. Therefore, we examined the effect of ATRA on the protein expression levels of epithelial (E‐cadherin) and mesenchymal (N‐cadherin) markers in HepG2 cells. ATRA treatment significantly inhibited the expression of EMT markers. Given our earlier finding that HCC migration may be controlled by CSTB via EMT, we assessed the impact of ATRA on CSTB protein expression. ATRA significantly downregulated CSTB expression. Crucially, overexpression of CSTB in HepG2 cells partially rescued the tumour‐suppressive effects of ATRA. These findings—the suppression of malignant phenotypes by ATRA and their partial reversal by CSTB overexpression—were consistently validated in both in vitro and in vivo models. Therefore, our experimental results demonstrate that ATRA inhibits HCC growth and metastasis by downregulating CSTB.

Following the confirmation of ATRA‐mediated CSTB suppression, proteomic sequencing identified cytochrome b (CYTB) as a downstream target of CSTB. CYTB is the only protein in complex III (CIII) encoded by a mitochondrial DNA (mtDNA) gene. CIII is a major site of reactive oxygen species (ROS) generation [38, 39]. Consistent with its identification as a CSTB target, CSTB overexpression increased CYTB expression, modulated CIII activity, and elevated ROS levels. CIII plays a critical role in the tricarboxylic acid (TCA) cycle and oxidative phosphorylation, driving ATP production. Impaired CYTB function leads to CIII dysfunction, resulting in excessive free radical generation and ultimately causing mitochondrial respiratory defects [40]. In our study, ATRA treatment significantly downregulated CYTB expression. Concomitantly, ATRA induced mitochondrial membrane potential dissipation, suppressed CIII activity, and reduced cellular ATP production in HepG2 cells. Crucially, CSTB overexpression partially reversed these ATRA‐induced impairments in mitochondrial function. This rescue effect strongly indicates that ATRA likely impairs mitochondrial function through the CSTB/CYTB axis.

Undoubtedly, our study has several limitations. First, the selection of only HepG2 cells based on database analysis potentially compromises the comprehensiveness of our findings. We acknowledge that the use of a single HCC cell line limits the generalisability of our results. Future studies should include additional HCC cell lines (e.g., Huh7, MHCC97H) or patient‐derived organoids to validate the role of the CSTB/CYTB axis across heterogeneous HCC models. Second, the precise molecular mechanisms by which ATRA regulates CSTB transcription and subsequently CYTB expression remain incompletely elucidated and require further investigation. Finally, the observation that CSTB overexpression only partially reversed the tumour‐suppressive effects of ATRA suggests that ATRA likely exerts its anti‐HCC activity through mechanisms beyond the CSTB/CYTB axis. Future studies are warranted to elucidate these alternative pathways.

## 5 Conclusion

Our findings demonstrate that CSTB plays a critical role in hepatocellular carcinoma (HCC) progression. All‐trans retinoic acid (ATRA) suppresses HCC progression by targeting the CSTB/CYTB axis, thereby inhibiting mitochondrial function in HCC cells. This study validates the tumour‐suppressive efficacy of ATRA in HCC and provides novel insights into its underlying mechanism, potentially holding important clinical implications for HCC therapy.

## Author Contributions

Jing Sun: writing – review and editing, writing – original draft, investigation, formal analysis, data curation, conceptualisation. Jian Zheng: visualisation, formal analysis, conceptualisation. Weiyi Zhu: visualisation, data curation, conceptualisation. Yang Bi: writing – review and editing. Yun He: writing – review and editing, supervision, project administration, funding acquisition.

## Funding

This work was sponsored by Natural Science Foundation of Chongqing, China (CSTB2024NSCQ‐MSX0486).

## Ethics Statement

Verbal consent was obtained from all participants. This study was approved by the Ethics Committee of Chongqing Medical University. The animal study was conducted according to the Guidelines for the Laboratory Animal Use and Care Committee of the Ministry of Health, China and the Ethics Committee on Animal Research of Chongqing Key Biomedical Laboratory (IACUC Issue No: CHCMU‐IACUC20230117001).

## Conflicts of Interest

The authors declare no conflicts of interest.

## Data Availability

The data used in the study analyses can be made available by the corresponding author on reasonable request.
